# Nose-to-brain delivery of borneol modified tanshinone IIA nanoparticles in prevention of cerebral ischemia/reperfusion injury

**DOI:** 10.1080/10717544.2021.1943058

**Published:** 2021-06-28

**Authors:** Luting Wang, Lin Xu, Junfeng Du, Xiao Zhao, Mei Liu, Jianfang Feng, Kaili Hu

**Affiliations:** a Institute of Interdisciplinary Integrative Medicine Research, Murad Research Center for Modernized Chinese Medicine, Shanghai University of Traditional Chinese Medicine, Shanghai, People’s Republic of China; b Shanghai Institute of Materia Medica, Chinese Academy of Sciences, Shanghai, People’s Republic of China; c Institute of Interdisciplinary Integrative Medicine Research, The Center for TCM Standardization, Shanghai University of Traditional Chinese Medicine, Shanghai, People’s Republic of China; d School of Pharmacy, Guangxi University of Chinese Medicine, Nanning, People’s Republic of China

**Keywords:** PEG–PLGA nanoparticles, borneol, tanshinone IIA, intranasal delivery, cerebral ischemia/reperfusion injury

## Abstract

Targeted treatment of cerebral ischemia/reperfusion injury (CIRI) remains a problem due to the difficulty in drug delivery across the blood–brain barrier (BBB). In this study, we developed Bo-TSA-NP, a novel tanshinone IIA (TSA) loaded nanoparticles modified by borneol, which has long been proved with the ability to enhance other drugs’ transport across the BBB. The Bo-TSA-NP, with a particle size of about 160 nm, drug loading of 3.6%, showed sustained release and P-glycoprotein (P-gp) inhibition property. It demonstrated a significantly higher uptake by 16HBE cells *in vitro* through the clathrin/caveolae-mediated endocytosis and micropinocytosis. Following intranasal (IN) administration, Bo-TSA-NP significantly improved the preventive effect on a rat model of CIRI with improved neurological scores, decreased cerebral infarction areas and a reduced content of malondialdehyde (MDA) and increased activity of superoxide dismutase (SOD) in rat brain. In conclusion, these results indicate that Bo-TSA-NP is a promising nose-to-brain delivery system that can enhance the prevention effect of TSA on CIRI.

## 1. Introduction

Nowadays, ischemia stroke remains one of the leading causes of disability and death worldwide (Wu et al., 2018). Cerebral ischemia/reperfusion injury (CIRI) happens when ischemia stroke patients are treated with rapid brain–blood reperfusion (Turner et al., 2013; Cuartero et al., 2016), which is the standard treatment for ischemia stroke with no other effective replacement. Hence, it is very important to find effective therapies for CIRI (Cui et al., 2016). The mechanism of CIRI involves toxicity of excitatory amino acid, Ca^2+^ overload (Fan et al., 2018), NO injury, mitochondrial damage, massive production of oxygen free radicals, immunoinflammatory injury, and apoptosis (Yang et al., 2016), which do not exist in isolation but are inseparable and interrelated and preventing these processes can be useful in decreasing the neural damage and promoting the recovery of cerebral ischemia patients. However, delivery of drugs to the central nervous system (CNS) has always been a challenge, mostly due to the presence of the BBB (Peluffo et al., 2015). To overcome this barrier, various brain targeting strategies including cell-penetrating peptides modification, nanoparticle (NP) encapsulation, intranasal (IN) delivery, P-glycoprotein (P-gp) inhibition and disruption of BBB were explored by researchers to improve brain disease therapy (Pasha & Gupta, 2010). As a noninvasive method for brain delivery, IN administration could bypass the BBB to allow therapeutic substances direct access to the CNS (Padowski & Pollack, 2010; Fazil et al., 2012; Bhattamisra et al., 2020). After IN administration, drug molecules can transport through the olfactory mucosa, olfactory nerve and trigeminal nerve to CNS (Lochhead & Thorne, 2012). Hence, IN might provide a faster and specific therapeutic effect for brain disease like CIRI (Illum, 2002; Gao, 2016; Yang et al., 2020).

TSA is the major active ingredient of a Traditional Chinese Medicine (TCM) *Salvia miltiorrhiza*, which has been widely used for the treatment of cerebrovascular diseases (Han et al., 2008). Modern clinical and pharmacological studies have shown a variety of activities of TSA such as significant inhibition of the degree of peroxidation, decrease the toxicity of excitatory amino acid, inhibit Ca^2+^ overload, decrease NO release, inhibit mitochondrial damage, decrease oxygen free radicals level, regulate the immunoinflammatory process, and inhibit apoptosis (Dong et al., 2018). Besides the notable curative effects for cardiovascular and cerebrovascular diseases, TSA also indicates various activities that might be effective in protection for CIRI (Tang et al., 2010; Liu et al., 2010a, 2011). However, due to the poor solubility of TSA, rapid plasma clearance, and P-gp efflux, it is difficult to pass through the blood–brain barrier (BBB), which greatly limits its therapeutic effect on CIRI. In this study, polymeric NPs were chosen to improve the brain targeting of TSA after IN administration. The variability of polymer carrier can give drug delivery system many new characteristics. Polyester materials are widely used because of their biodegradability, good biocompatibility, and safety. Free design of polymer chain length can produce different particle size, drug delivery capacity, and biological effects.

Borneol (Bo), a guiding drug in TCM, is usually used as an adjuvant component to facilitate the delivery of other components in TCM (e.g. in compound Danshen dropping pills) for the treatment of cardiovascular and cerebrovascular diseases (Lu et al., 2010). It was demonstrated that Bo could easily across the nasal mucosa, open up the BBB, and enhance the distribution of other drugs into brain (Wang et al., 2019). Recently, the investigation on effect of Bo on BBB transport of drug loading NPs also proved that Bo can guide NPs into the brain after intravenous injection (Yu et al., 2013). Our previous studies demonstrated the enhancing effects of Bo on NP delivery after IN administration, while whether these enhancing effects can actually improve brain disease therapy is still unknown (Wang et al., 2019). What is more interesting, this guiding drug Bo also was reported with pharmacological effects on the CNS (Cho et al., 2002; Ren et al., 2013; Yu et al., 2013) such as anti-inflammation, attenuating neuronal apoptosis, and comprehensively improve neurovascular unit function (Liu et al., 2011; Duan et al., 2019). Thus, in this study, we constructed a Bo-modified TSA loaded NP, and investigated its therapeutic effect on CIRI after IN delivery.

In this paper, to improve the brain delivery and protective effect of TSA on CIRI, the PEG–PLGA NPs with biodegradable property and good biocompatibility were synthesized by the W/O/W double emulsion-solvent evaporation method and then modified by Bo. Bo-TSA-NP was prepared by chemical conjugation of bornylamine to the succinimide group of N-hydroxy succinimide–poly(ethyleneglycol)–poly(lactic-co-glycolic acid) (NHS–PEG–PLGA, Mw 41,173) on the surface of methoxy poly(ethyleneglycol)–poly(lactic-co-glycolic acid) (mPEG–PLGA, Mw 34,986) NPs, while Bo-loaded-TSA-NP was prepared by physical mix of Bo into the NPs. The formulation was first optimized by a response surface method and then the prepared NPs were characterized in aspects of morphology, particles size, zeta potential, drug loading, and *in vitro* drug release profile. The brain delivery effect and transport mechanism of Bo-NP was investigated *in vitro* by a 16HBE cell model and compared to Bo-loaded-NP. In addition, the protective effect of Bo-TSA-NP on CIRI was evaluated *in vivo* in a rat model of focal CIRI.

## 2. Materials and methods

### 2.1. Materials

mPEG–PLGA was purchased from Jenkem Technology Co., Ltd. (Beijing, China). Coumarin-6, dimethyl sulfoxide, (R)-(+)-bornylamine, and verapamil (≥97%) were purchased from Sigma-Aldrich (St. Louis, MO). MCAO and TTC were obtained from Cinontech Co., Ltd. (Beijing, China). Sodium dodecyl sulfate (electronic pure) were obtained from Yu Yun Technology Co., Ltd. (Hong Kong, China). Tanshinone IIA (TSA) standard (>98%), Bo, Tween 80, sodium chloride, calcium chloride, sodium bicarbonate, potassium chloride, methanol, and dichloromethane (analytical grade) were purchased from Sinopharm Group Chemical Reagent Co. Ltd. (Shanghai, China). Methanol and acetonitrile (HPLC grade) were purchased from Honeywell (Charlotte, NC). Twenty-four-well plates were purchased from Corning (Corning, NY). Rhodamine 123 (R-123) was purchased from Aladdin Reagent Co., Ltd. (Shanghai, China).

16HBE cells were obtained from Cell Bank of Chinese Academy of Sciences (Shanghai, China). DMEM medium, fetal bovine serum (FBS), trypsin, and non-essential amino acids were purchased from Thermo Scientific (Shanghai, China). The BCA kit was purchased from Shenneng Bocai Company (Shanghai China).

Male Sprague-Dawley (SD) rats (230 ± 20 g, ♂) were obtained from Shanghai Slack Animal Co. Ltd. (Shanghai, China) and maintained at 22 ± 2 °C on a 12 h light–dark cycle with access to food and water *ad libitum*. The animals used for the experiment were treated according to the protocols evaluated and approved by the ethical committee of Shanghai University of Traditional Chinese Medicine.

### 2.2. Synthesis of Bo–PEG–PLGA

The NHS–PEG–PLGA and mPEG–PLGA polymers were synthesized by ring opening polymerization as described previously (Zhang et al., 2004). The Bo modified Bo–PEG–PLGA polymer was synthesized by incubating of NHS–PEG–PLGA with bornylamine at a molar ratio of 9:1 at room temperature for 24 h. After purification by methanol precipitation, the obtained Bo–PEG–PLGA was identified by NMR and FTIR.

### 2.3. Formulation optimization

#### 2.3.1. Single factor investigation

The dosage of TSA (mg), ultrasound power (W), and the amount of polymer (mg) in the preparation of NPs were investigated by a single factor experiment with particle size and drug loading capacity (DLC) of the NP as response index. The factors have major effects on the size and DLC of NP will be chosen for further formulation optimization.

#### 2.3.2. Central composite design and response surface methodology

According to the results of single factor investigation, two factors which have significant influence on the properties of NP were selected: dosage of TSA and amount of polymer (mg). A two factors and five levels star design was then used based on a two-level factorial design with extremum points and center points to find the optimal formulation. The codes are +1, +*α*, and 0, where *α*=(*F*)1/4, *F* = 2*k* (*k* is the factor number), and *α* = 1.414. After determining the maximum (+*α*) and the minimum (–*α*) of each factor according to the results of the pre-experiment, the level of (+1, 0) is arranged according to the principle that the difference between any two physical quantities is proportional to the difference between the corresponding codes. The code values and corresponding specific values of different factors are shown in Table 1. The experimental design is shown in Table 2.

**Table 1.** Levels of independent variables in coded and specific forms.

	Level				
	–1.414	–1	0	+1	+1.414
*X*_1_	0.3	0.55	1.15	1.75	2.0
*X*_2_	10	14.39	25	35.61	40

*X*_1_: dosage of TSA; *X*_2_: amount of polymer.

**Table 2.** Experimentally determined values of different dependent variables.

	Factor^a^		Dosage (%)	Size (nm)
	*X*_1_ (mg)	*X*_2_ (mg)		
1	1.75	35.61	3.373	148.2
2	1.75	14.39	6.099	1180.2
3	0.55	35.61	1.217	140.6
4	0.55	14.39	2.604	2380.1
5	2.0	25	6.436	412.5
6	0.3	25	1.195	137.6
7	1.15	40	2.246	152.1
8	1.15	10	7.315	826.5
9	1.15	25	3.313	173.1
10	1.15	25	3.295	164.9
11	1.15	25	3.255	165.5
12	1.15	25	3.317	164.9
13	1.15	25	3.332	165.8

^a^ *X*_1_: dosage of TSA (0.3–2.0 mg); *X*_2_: amount of polymer (10–40 mg).

### 2.4. Preparation of nanoparticles

TSA-loaded PEG–PLGA nanoparticles (TSA-NP) were prepared by double emulsion-solvent evaporation method using TSA as a model drug. After dissolving 28 mg mPEG–PLGA to 1 mL of 1.4 mg/mL TSA-containing dichloromethane solution, 50 μL of ultrapure water was added and the solution was probed for 30 s. Then, the solution was added to a tube containing 2 mL 1% sodium cholate and further probed intermittently for 1 min. Finally, the solution was added to 20 mL of 0.5% sodium cholate, magnetically stirred for 30 min, and the organic solvent was removed by rotary evaporation. The Bo-loaded-TSA-NP was prepared by the same method except 5 µg Bo was added to the 1.4 mg/mL TSA solution before the first sonification. The Bo-TSA-NP was prepared by the same method but using 28 mg materials (Bo–PEG–PLGA:mPEG–PLGA = 1:20) instead of 28 mg of pure mPEG–PLGA.

### 2.5. Characterization of nanoparticles

#### 2.5.1. Transmission electron microscope observation

The TSA-NP, Bo-TSA-NP, and Bo-loaded-TSA-NP were negatively stained with 1–2% (w/v, pH 7.0) phosphotungstic acid, and their morphology was observed under transmission electron microscope.

#### 2.5.2. Particle size and zeta potential

After diluted with double-distilled water, the particle size and zeta potential of NPs were measured by the NICOMPZLS380 laser scattering particle size/zeta potential analyzer (PSS, Santa Barbara, CA) and the particle size distribution was observed.

#### 2.5.3. Entrapping efficiency (EE) and drug-loading capacity

One milliliter of the prepared NP was centrifugated (12,000 rpm, 30 min, 4 °C) and the precipitate was dissolved in 0.5 mL acetonitrile. For TSA loaded NPs, the solution was further diluted 20 times with methanol and 10 μL supernatant was used for HPLC detection of TSA after centrifugated. For coumarin-6 loaded NPs, the fluorescence intensity of coumarin-6 was determined by VARIOSKANFLASH full-wavelength scanner (Thermo, Waltham, MA). The concentration of TSA or coumarin-6 was calculated by a validated standard curve. EE and DLC were calculated according to the following equations:

(1) $$\mathrm{EE}(\%)=\frac{W1}{W}\times 100\%$$

(2) $$\mathrm{DLC}(\%)=\frac{W1}{\left(W1+W2\right)}\times 100\%$$

where *W*_1_ is the amount of coumarin-6 or TSA encapsulated in the NPs, *W*_2_ is the amount of the carrier material. *W* refers to the total amount of TSA or coumarin-6 added during preparation.

### 2.6. *In vitro* release

The *in vitro* release characteristics of NPs were investigated by centrifugal tube method (Zhang et al., 2004). TSA-NP was centrifuged at 12,000 rpm for 40 min at 4 °C, and the pellet was suspended with 100 mL of artificial nasal fluid containing 1% Tween-80 or PBS with 5% plasma. Each 1 mL of the solution was transferred into 1.5 mL centrifuge tubes. All the 54 tubes were put in a shaking bath at 37 °C, 100 rpm. At 0, 0.25, 0.5, 1, 2, 4, 8, 12, and 24 h after incubation, six tubes were withdrawn. Three of them were centrifuged at 4 °C, 12,000 rpm for 20 min, and the supernatant was diluted with acetonitrile and the amount of TSA was calculated by HPLC. Acetonitrile was added to the other three tubes to destroy the NPs to determine the total amount of TSA in the NPs. The cumulative release percentage of TSA was calculated according to the proportion of TSA released to the total amount of TSA at different time.

### 2.7. *In vitro* uptake of Bo-NP and Bo-loaded-NP by 16HBE cells

As a highly sensitive lipid soluble fluorescent dye, coumarin-6 is often used to study the *in vitro* and *in vivo* behavior of nano drug delivery systems. In this paper, coumarin-6 was used as a fluorescent probe to study the brain targeting effects of NPs intuitively. 16HBE cells which are human bronchial epithelioid cells and retain the natural functions of typical absorption epithelium are an attractive candidate as a model of the nasal mucosa epithelial. The 16HBE cells were seeded at a density of 10^5^/mL to 24-well plates and cultured for 24 h. Then, the cells were incubated with HBSS balance solution for 15 min at 37 °C, before HBSS diluted coumarin-6-NP, Bo-coumarin-6-NP and Bo-loaded-coumarin-6-NP were added (Gänger & Schindowski, 2018). After experiment, the cells were washed three times with 1 mL PBS before treated with 400 μL of 1% Triton-X 100 and the lysate was stored at −20 °C. One hundred milliliter of the lysate was used to determine the concentration of coumarin-6 by a Varioskan Using flash full-wavelength scanning multi-function reader, 20 μL of the lysate was used to determine the protein concentration of the cells by BCA kit. The effects of different modification methods, modification ratios, uptake times, NP concentrations, temperatures, and uptake inhibitors on 16HBE uptake of NPs were investigated.

### 2.8. Effects of Bo-NP and Bo-loaded-NP on the uptake of R-123 by 16HBE cells

16HBE cells were seeded at a density of 10^5^/mL onto 24-well plate. The next day, after pre-incubated with HBSS solution (37 °C) for 15 min, different concentrations (50, 100, and 200 ng/mL) of Bo-NP and Bo-loaded-NP were incubated with the cells for 15 minutes before R-123 solution was added to incubate for 1 h at 37 °C. The negative control group was incubated with PBS and 1.0 µg/mL verapamil solution was incubated as the positive control. After R-123 incubation, the uptake was terminated by cold PBS. The cells were washed for three times with PBS before treated with 400 µL 1% Triton-X 100, and the lysate was stored at −20 °C for further detection. The concentration of R-123 was determined by Varioskan Flash full-wavelength scanning multi-function reader with a validated method and the protein concentration of cells was determined by BCA kit.

### 2.9. Establishment of a rat focal CIRI model

SD rats were randomly divided into four groups (*n* = 8), which are the model group, TSA-NPs group and Bo-TSA-NP group. Three days after continuous nasal administration of NPs at TSA dose of 0.5 mg/kg (100 μL), a focal CIRI model was established at 1 h after the last administration. After anesthetized with 2% pentobarbital sodium, the bifurcation of the left carotid artery of rats was exposed, and the left common carotid artery (CCA) and internal carotid artery (ICA) were isolated and ligated. The external carotid artery (ECA) was isolated, and a 4-0 nylon suture was inserted from the ECA to the ICA to occlude the origin of the middle cerebral artery (MCA). After 2 h of middle cerebral artery occlusion (MCAO), the suture was removed to allow reperfusion of the ischemic area via the CCA. The behavior of the rats was evaluated according to Longa’s score criteria (Lochhead & Thorne, 2012). Three animals in each group were randomly selected for the observation of cerebral infarction and the calculation of the infarction percentage. The remaining five rats were used for the determination of malondialdehyde (MDA) content and superoxide dismutase (SOD) activity in the brain tissue.

This section complies with the National Institutes of Health Laboratory Animal Care and Use Guidelines.

### 2.10. Cerebral infarction

After the neurological assessment at 24 h after MCAO, the rats were euthanized under deep anesthesia and the brain was quickly removed and sectioned into 2 mm-thick coronal slices. The slices were stained with 2% 2,3,5-triphenyltetrazolium chloride (TTC) at 37 °C for 30 min and then immersed in 4% buffered paraformaldehyde solution for fixation. Infarct areas were first measured using image analysis software and then compiled to obtain the infarct volume (mm^3^) per brain. The percentage of infarction (infarct ratio) was calculated from the data of infarct volume and total coronal section using following formula:

(3) $$\text{Infarct ratio }(\%)=\frac{\text{pale part }(g)}{\left[\text{pale part }(g)+\mathrm{non}-\text{pale part }(g)\right]}\times 100\%$$

### 2.11. Brain malondialdehyde and superoxide dismutase detection

After the neurological assessment, the rats were anesthetized and the lower brain stem, cerebellum and olfactory bulb were removed from the brain. The left hemisphere was weighed and added to ice-cold physiological saline at a ratio of 1:9 (w/v) to prepare a 10% brain tissue homogenate. After centrifugation at 2500 rpm for 10 min, the supernatant was collected. The contents of MDA and SOD in the supernatant as well as the content of tissue protein were determined by Nanjing Institute of Bioengineering (Nanjing, China) according to the protocols.

### 2.12. Statistical analysis

The statistical analysis was performed using GraphPad Prism from GraphPad Software (La Jolla, CA). Data were reported as the mean ± SD. Data were compared using one-way ANOVA and unpaired two-tailed *t* tests and *p* values less than .05 were considered to be statistically significant.

## 3. Results

### 3.1. Characterization of Bo–PEG–PLGA

**Figure 1.**
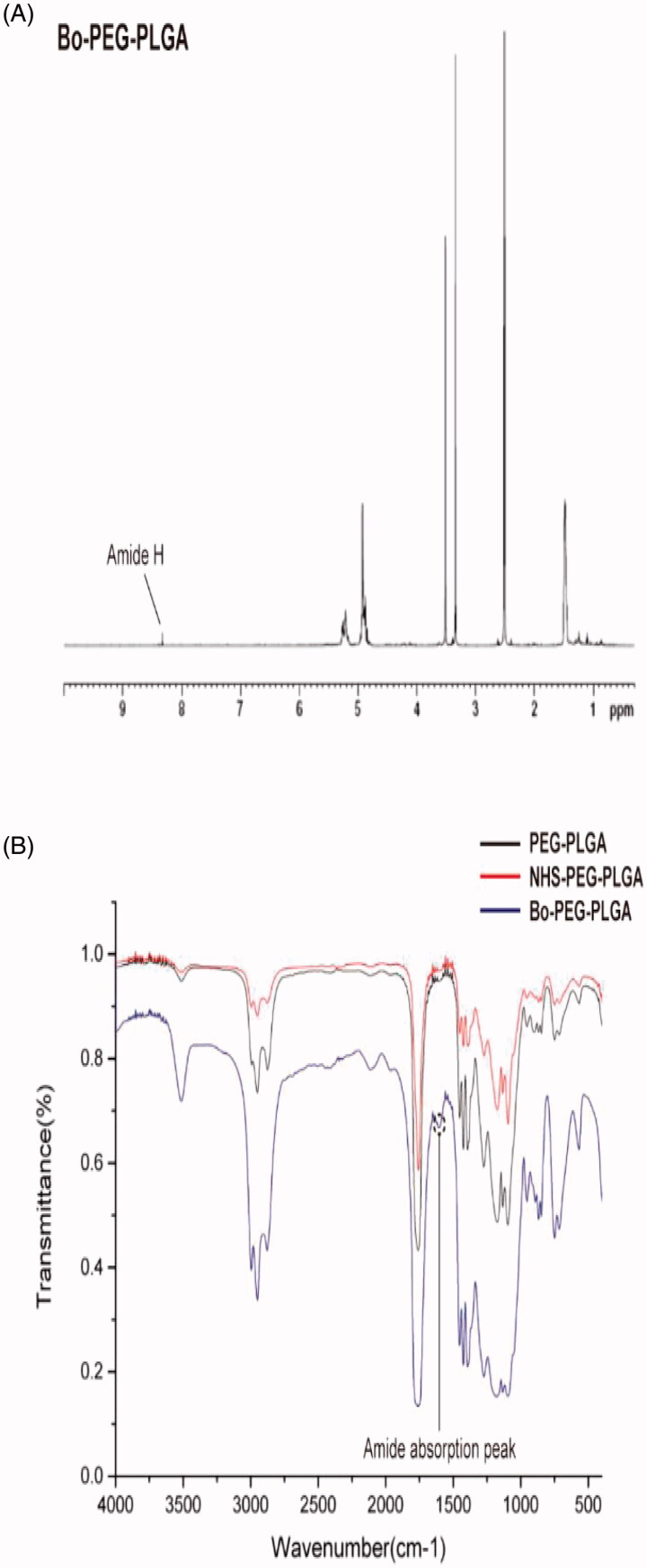
Structure identification of Bo–PEG–PLGA (A)^1^H NMR spectrum in DMSO and (B) FTIR spectrum.

The FTIR and ^1^H NMR spectrums of the synthesized Bo–PEG–PLGA are shown in Figure 1. The synthesized Bo–PEG–PLGA exhibits an amide hydrogen signal at about 8.3 ppm while there is no such signal in the ^1^H NMR of the starting material. In addition, the characteristic absorption peak of the amide at FTIR 1600 cm^−1^ indicates that the product has a new amide bond, which linked the polymer PEG–PLGA backbone and the bornylamine. Both of these proved the successful synthesis of the Bo–PEG–PLGA and the calculated molecular weight of the polymer was 43,743.

### 3.2. Formulation optimization of Bo-TSA-NP and Bo-loaded-TSA-NP

**Figure 2.**
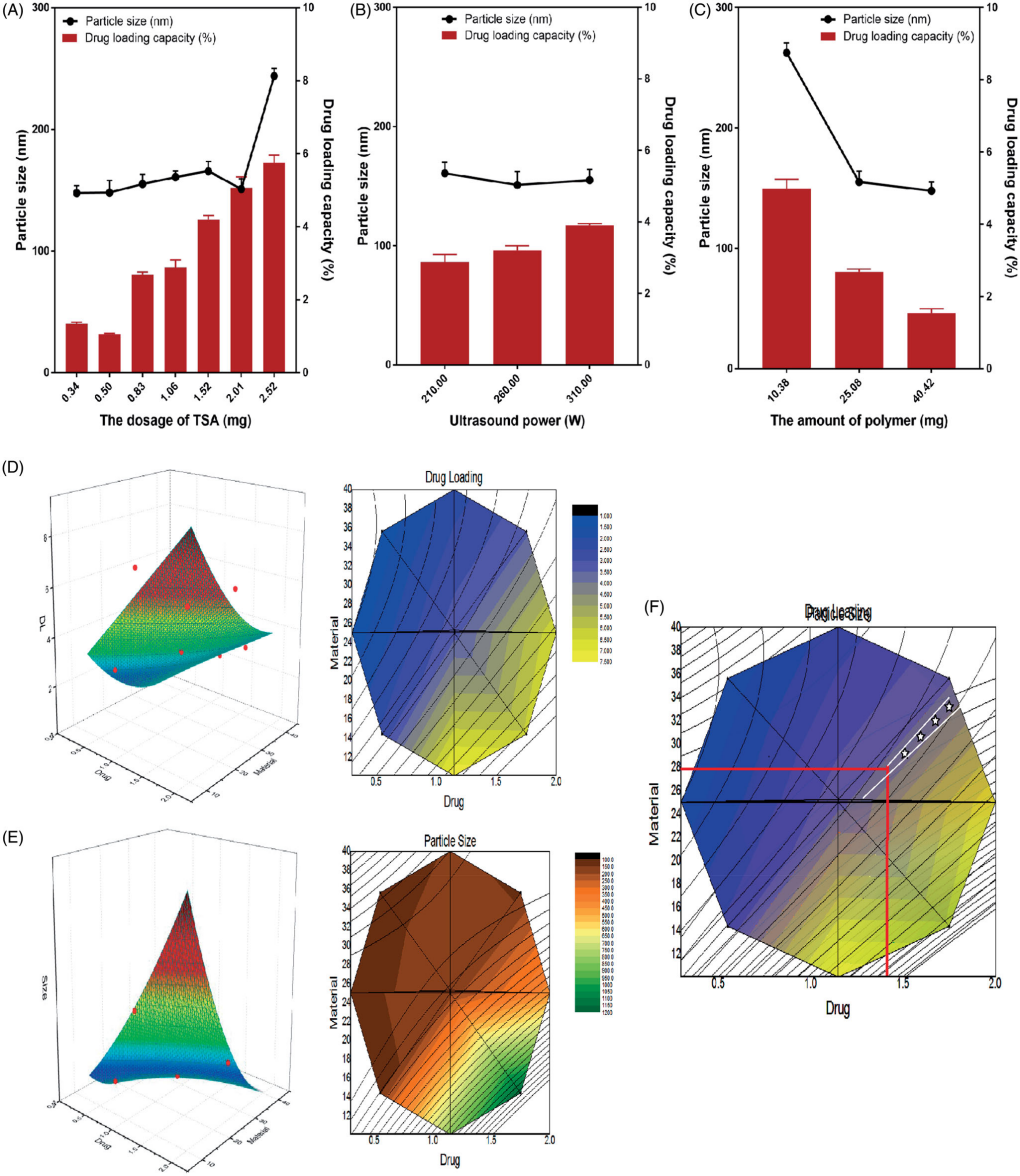
Optimization of the formulation of TSA-NP. The effect of (A) the dosage of TSA, (B) ultrasound power, and (C) the amount of polymer on the size and DLC of the TSA-NP; response surface plot and contour plot showing the effect of the dosage of TSA and the amount of polymer added on the response of (D) DLC and (E) particle size; (F) the overlapping of the contour plots of D and E.

As shown in Figure 2, the results of the single factor experiments indicated that with the increase of the dosage of TSA, the particle size and drug loading of the NPs increased gradually (Figure 2(A)). While with the increase of the amount of polymer, the particle size, and drug loading of the NPs decreased gradually (Figure 2(B)). The ultrasonic power has much less effect on the two-response index (Figure 2(C)). Therefore, the dosage of TSA and the amount of the polymer were chosen to carry on the following central composite-response surface experiment. In the response surface experiment, each effect surface has its own superior regions, and the superior conditions selected by multiple effects can further reduce the range of the better regions by superposition. With Origin pro 8.0 as the statistical software for prescription optimization, the three-dimensional response surface and two-dimensional contour map of each effect on two factors were described according to binomial fitting equation in Table 3. Each response surface has its own optimal region. The dosage of TSA (*X*_1_) and the amount of PEG–PLGA (*X*_2_) and their interaction on the drug loading (*Y*_1_) are shown in Figure 2(D). The results show that when the dosage of TSA is constant, the drug loading of the NPs decreases with the increase of the amount of PEG–PLGA. When the amount of PEG–PLGA is constant, the drug loading of the NPs increases with the increase of the dosage of TSA. The dosage of TSA and the amount of PEG–PLGA and their interaction on the particle size (*Y*_2_) are shown in Figure 2(E). The results show that when the dosage of TSA is constant, the NP size decreases with the increase in the amount of PEG–PLGA. When the amount of PEG–PLGA is constant, the particle size of the NPs increases with the increase of the dosage of TSA. The optimal conditions selected by multiple effects can be further reduced by superposition. Through the contour lines of each effect surface described by origin Pro 8.0 software, the ideal and better area can be obtained by superposition. The results are shown in Figure 2(F). According to binomial equation of each effect value, considering the actual situation of preparation, we get the best prescription in overlap area: *X*_1_=1.4 mg, *X*_2_=28 mg.

**Table 3.** Coefficients of regression equations for the responses.

Dependent variable (responses, *Y*)	Regression coefficients						
	*b*_0_	*b*_1_	*b*_2_	*b*_3_	*b*_4_	*b*_5_	*R*_2_
Drug loading (*Y*_1_)	4.740	3.997	−0.290	0.016	0.004	−0.053	0.919
Particle size (*Y*_2_)	377.955	930.488	–49.993	167.711	1.463	–40.516	0.959

Based on the optimized TSA-NP formulation, the chemical modified Bo-TSA-NP were prepared by the basic formulation with 28 mg of polymer (Bo-PLGA-PEG:PLGA-PEG = 1:20) and 1.4 mg TSA. The Bo-loaded-TSA-NP was prepared by the basic formulation with 28 mg PLGA–PEG, 5.0 μg Bo, and 1.4 mg TSA.

### 3.3. Characterization of Bo-loaded-TSA-NP and Bo-TSA-NP

**Figure 3.**
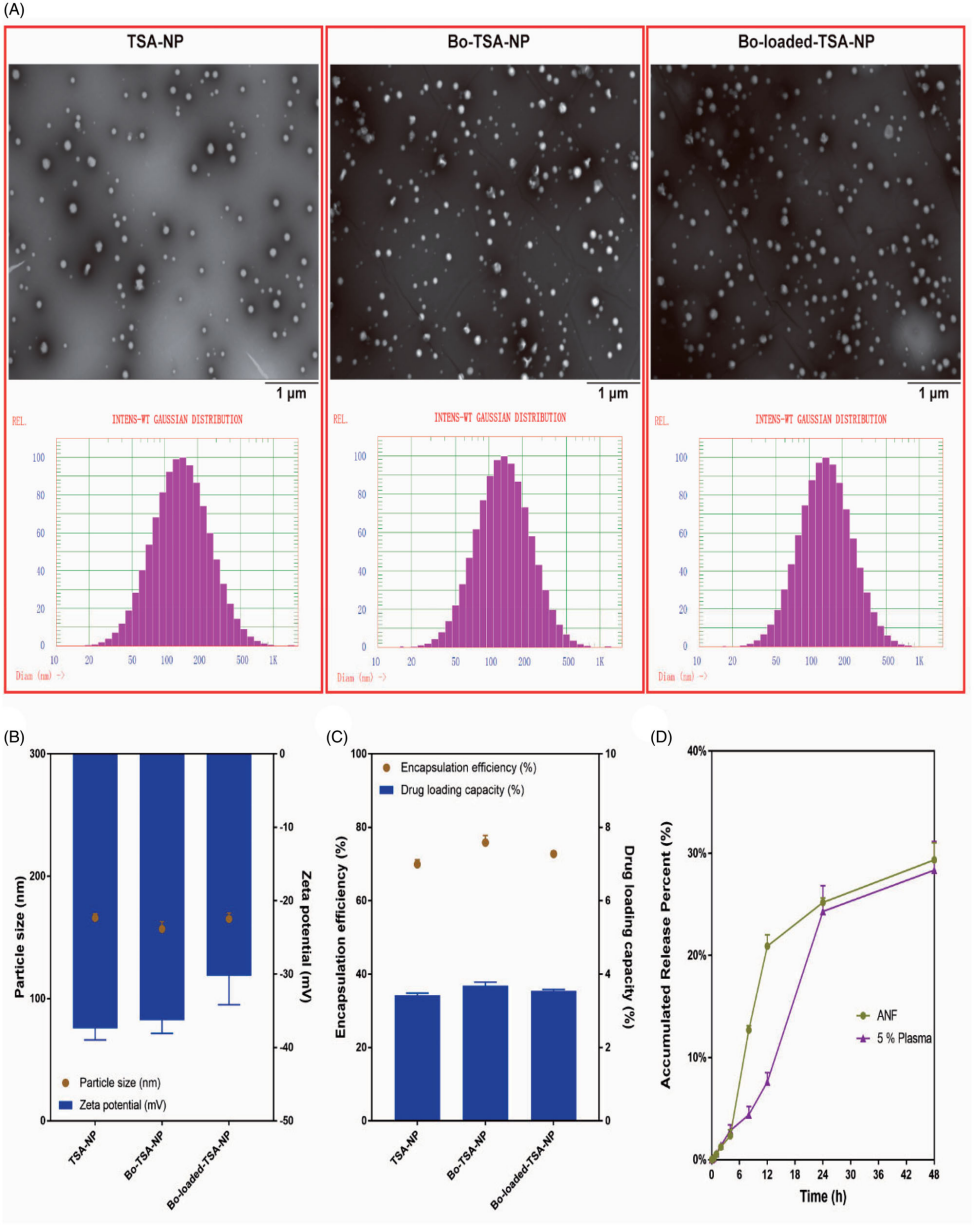
Characterization of Bo-loaded-TSA-NP and Bo-TSA-NP. (A) TEM photograph and size distribution; (B) size and zeta potential; (C) EE and DLC; (D) *in vitro* release of TSA in 37 °C ANF and 37 °C 5% plasma.

The morphologies of the Bo-loaded-TSA-NP, Bo-TSA-NP, and TSA-NP are all round with particle sizes of around 160 nm and zeta potentials of about −36 mV (Figure 3(A,B)). The modification of Bo does not have significant effect on the particle size and surface charge of NP. The EE and DLC detected by HPLC method of the three NPs were all about 70% and 3.6% (Figure 3(C)). Similar EE and relatively high DLC of the NPs showed good DLC of the NPs. The cumulative release of TSA-NP in artificial nasal fluid and 5% plasma was about 30% at 48 h, which indicated that it had a sustained release effect in ANF and 5% plasma *in vitro* (Figure 3(D)).

### 3.4. *In vitro* uptake of Bo modified NPs by 16HBE cells

**Figure 4.**
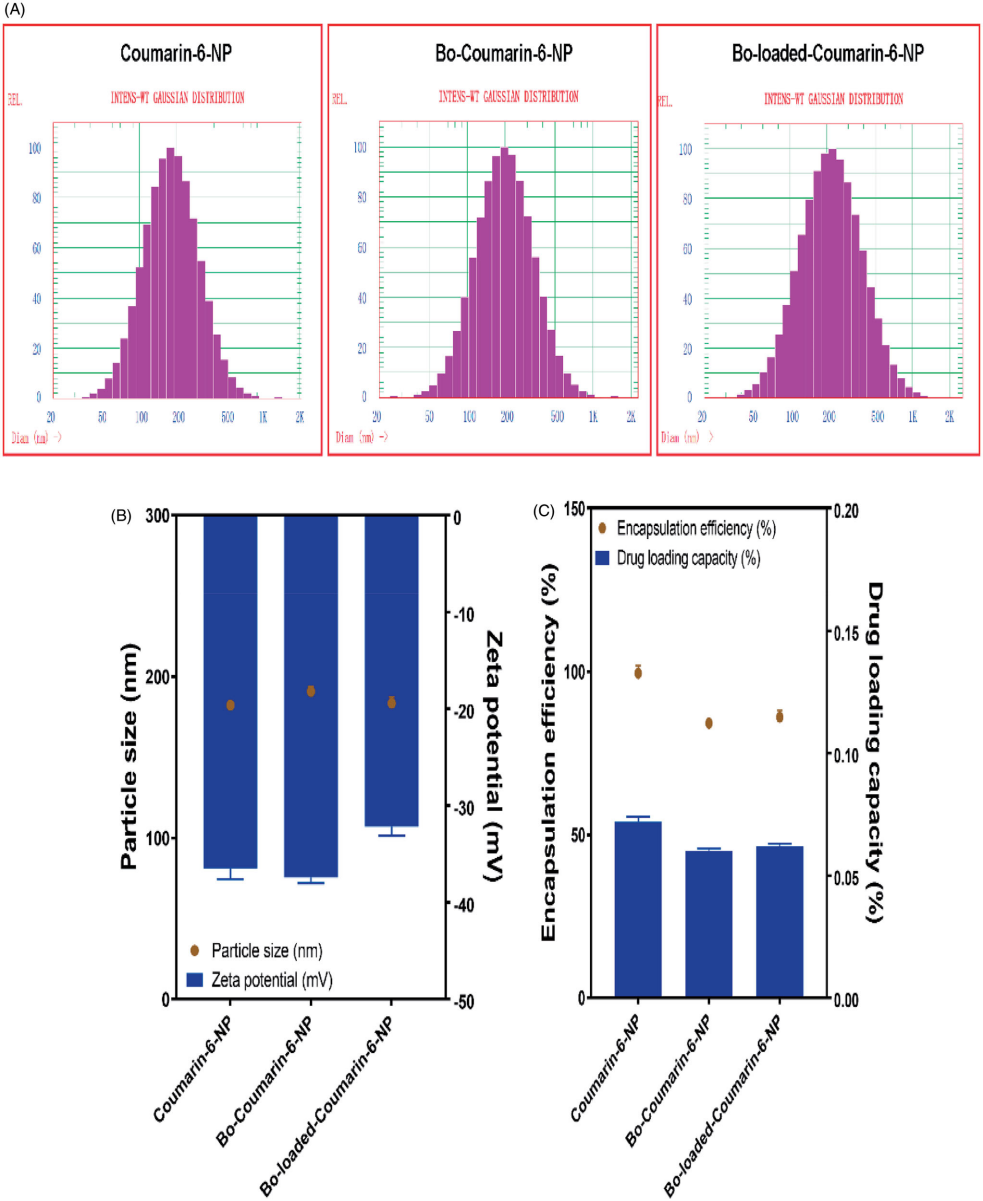
Characterization of coumarin-6-NP, Bo-coumarin-6-NP, and Bo-loaded-coumarin-6 NP. (A) Size distribution; (B) size and zeta potential; (C) EE and DLC.

To evaluate the brain delivery property of Bo modified NPs after IN delivery, coumarin-6 was used as a florescent probe and the 16HBE cells were used as a model to simulate the nasal mucosa. The coumarin-6 loaded NPs were prepared with the optimized formulations using 1.4 mg coumarin-6 instead of TSA. The particle sizes of the coumarin-6-NP, Bo-coumarin-6-NP, and Bo-loaded-coumarin-6-NP were all around 180 nm and with zeta potentials of about −35 mV. The DLC of the NPs were all about 0.065% (Figure 4(A–C)).

**Figure 5.**
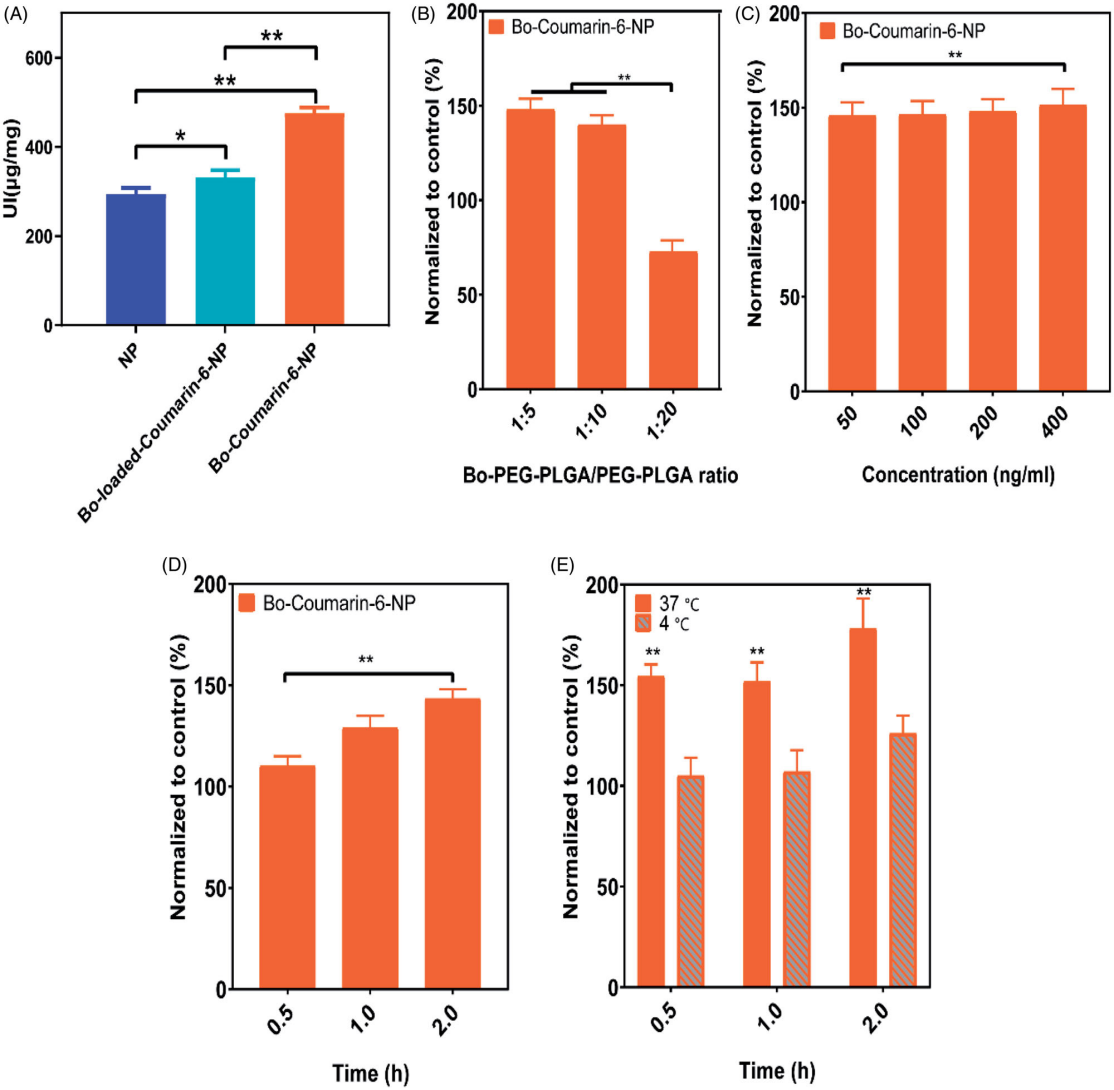
*In vitro* uptake of coumarin-6-NP, Bo-coumarin-6-NP, and Bo-loaded-coumarin-6 NP by 16HBE cells. (A) Uptake index of coumarin-6-NP, Bo-coumarin-6-NP, and Bo-loaded-coumarin-6-NP in 16HBE cells; effects of different (B) Bo–PEG–PLGA/PEG–PLGA ratio, (C) NP concentration, (D) incubation time, and (E) temperature on the uptake index of Bo modified NPs by 16HBE cells (compared to un-modified coumarin-6 NP). *p< .05; **p< .01.

**Figure 6.**
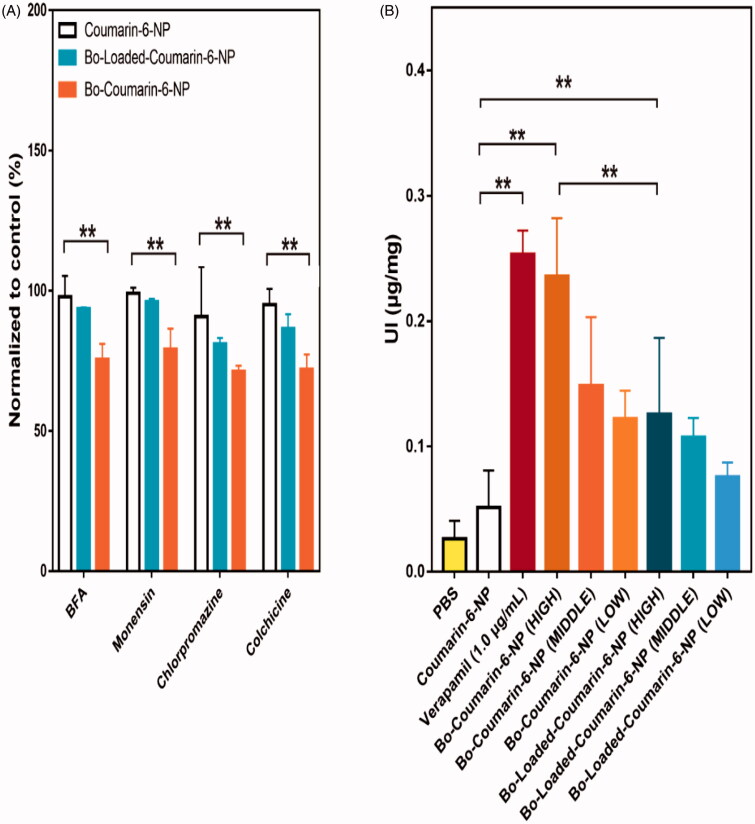
(A) Effect of different inhibitors on the uptake of different NPs by 16HBE cells; (B) effects of the different nanoparticles formulations on the uptake of R123 by 16HBE cells (LOW, MIDDLE, and HIGH indicates nanoparticle concentration of 0.25 μg/mL, 0.5 μg/mL, and 1 μg/mL, respectively). **p< .01.

The cell uptake results showed that compared with Bo-loaded-NP, the Bo-NP has a significantly enhanced uptake, suggesting that chemical modification method may have better IN delivery effect (Figure 5(A)). The uptake amount of Bo-NP by the cells increases with the proportion of the modified polymer in the formulation and reach a plateau at the 1:10 ratio (Figure 5(B)). The uptake of Bo-NP increased with the increase of NP concentration and the incubation time, showing a dose and time dependent manner (Figure 5(C,D)). In addition, the uptake of Bo-NP by 16HBE cells was also significantly affected by the uptake temperature with a more prominent uptake at 37 °C than at 4 °C. To further elucidate the uptake mechanism of Bo-NP by the 16HBE cells, we investigated the effects of different cell transport inhibitors on the uptake process. The results indicated that unlike Bo-loaded-NP, the uptake of the Bo-NP was significant inhibited by colchicine, chlorpromazine, monensin, and brefeldin A (BFA) (Figure 6(A)). These results suggest that unlike Bo-loaded-NP, Bo-NP can be untaken by endocytosis and pinocytosis which is an energy-dependent processes involving the Golgi apparatus and lysosomes.

The *in vitro* uptake results demonstrated that chemically modified Bo has better brain delivery effects than physically modified Bo. To investigate the reason for this disparity, we further studied the uptake mechanism of the two kinds of NPs. The results suggested the Bo-NP is more actively involved in endocytosis and pinocytosis which may be one reason of its improved uptake by the 16HBE cells. In addition, Bo-NP well retains the effect of Bo on P-gp inhibition, which may be another reason for its higher uptake.

### 3.5. Protective effects of Bo-TSA-NP on a rat model of CIRI after intranasal administration

To further explore the pharmacodynamics of the Bo-TSA-NP, a rat model of CIRI was established and the protective effect of IN administrated Bo-TSA-NP was studied. Longa’s score calculated was 3.38 ± 0.74 for the TSA-NP group and 2.38 ± 0.74 for the Bo-TSA-NP group, suggesting that after IN administration, Bo-TSA-NP can significantly protect the neurological impairment caused by CIRI compared with TSA-NP (Table 4). This may be attributed to the better brain delivery property of the Bo-TSA-NP.

**Table 4.** Numbers of rats in each group with different neurological scores.

Group	Points^a^						Average
	0	1	2	3	4	5	
Model	0	0	0	1	3	4	4.38 ± 0.74
TSA-NP	0	0	1	3	4	0	3.38 ± 0.74*
Bo-TSA-NP	0	1	3	4	0	0	2.38 ± 0.748**

* Compared with model *p*<.05.

** Compared with model *p*<.01.

^a^ 0 points: normal nerve function; one point: mild neurological deficit (flexion of the left forelimb when the tail is lifted); two points: moderate neurological deficit (turning to the left in a circle when walking); three points: moderate neurological deficit (tilted to the left); four points: no spontaneous release, decreased consciousness; five points: death related to ischemia.

**Figure 7.**
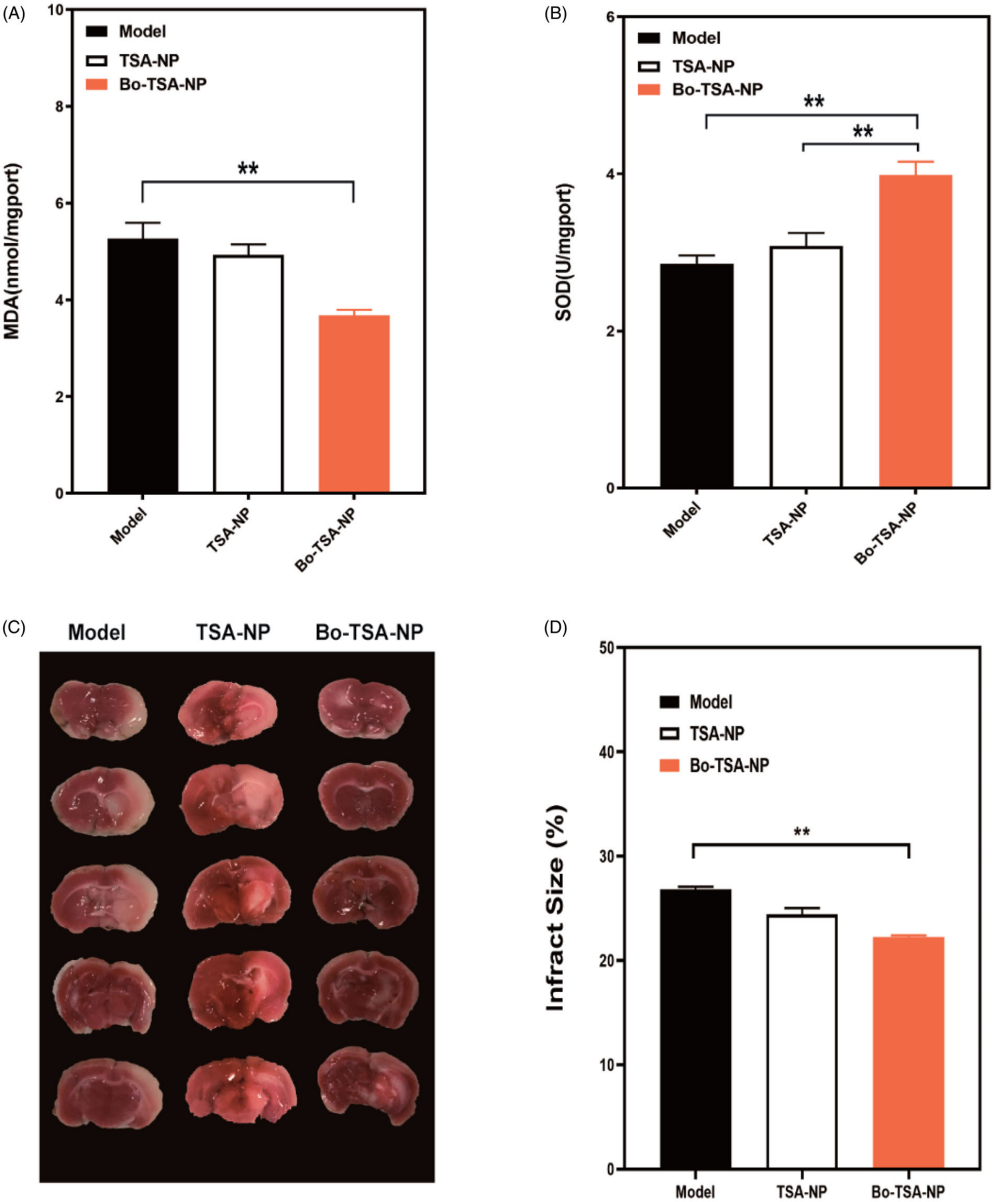
Protective effects of Bo-TSA-NP on a rat model of CIRI after intranasal delivery. Effects of Bo-TSA-NP on (A) MDA and (B) SOD concentrations in rat brain tissues after CIRI; (C) photograph of sectioned brain after the experiment and (D) calculated brain infract size. **p<.01.

The results of triphenyltetrazolium chloride staining showed that the boundary between the infarcted area (white) and the surrounding normal brain tissue (red) was clear (Figure 7(C)). The Bo-TSA-NP can better prevention the formation of the cerebral infarction. The calculated percentage of cerebral infarction of Bo-TSA-NP group was also significantly lower than that of TSA-NP group (*p*<.01) (Figure 7(D)). All these results suggested that Bo-TSA-NP can significantly ameliorate the cerebral infarction in focal cerebral ischemia–reperfusion rats.

We further detected the content of MDA and the activity of SOD in brain tissues. The results showed that the MDA content was significantly increased and the activity of SOD was significantly decreased in the model group and TSA-NP group (*p*<.05). While Bo-TSA-NP group showed a prominently decreased content of MDA and a significantly increased SOD activity (*p*<.01) compared with TSA-NP group. It suggested that the Bo-TSA-NP can promote the effects of TSA to reduce the degree of cerebral peroxidation and to scavenge the oxygen free radicals in the brain of cerebral ischemia reperfusion rats.

## 4. Discussion

To improve the brain delivery and protective effect of TSA on CIRI, the Bo modified TSA loaded NPs, Bo-TSA-NP, and Bo-loaded-TSA-NP, were prepared based on an optimized formulation of TSA-NP. During the optimization of the formulation of TSA-NP, the central composite design (CCD) method was used among the standard designs used in response surface methodology (RSM), because of its high efficiency with respect to the number of required runs (Longa et al., 1989). Besides, CCD also has high test accuracy, and is suitable for multi-factor and multi-level experiments (Liu et al., 2016). The biggest advantage of this CCD-RSM design method is that it can predict untested tests, but at the same time, it must have a wealth of expertise and certain pre-experiments to determine the scope of each factor. By using the CCD-RSM, we optimized the formulation of TSA-NP, based on which the optimized Bo-TSA-NP and Bo-loaded-TSA-NP were prepared. We used physical encapsulation (Bo-loaded-TSA-NP) and chemical conjugation (Bo-TSA-NP) to modify Bo on the NPs to investigate the effects of different modification methods on the brain targeting effect.

To overcome the low bioavailability of tanshinones, many methods have been used to improve the solubility of them, and NPs have shown better results than other formulations (Liu et al., 2010b). Particle size and surface properties of drug particles play a vital role in nasal drug delivery (Cai et al., 2016). The NPs prepared in this study have particle sizes of around 160 nm which facilitate endocytosis and are favorable for IN delivery. The stable surface charges indicated good stability and sustained release can further promote intracerebral delivery after IN administration. The DLC of the NPs were around 3.6% which are higher than the reported TSA-PLGA-NPs and a higher DLC is also a privilege for nasal delivery (Khan et al., 2017).

Bo is reported to exert its BBB penetration effects partly by its effects on P-gp inhibition (Fan et al., 2015; Zou et al., 2017). Therefore, we tested the effects of blank Bo modified NPs on the uptake of R-123 by 16HBE cells to investigate whether the Bo modified on the NP surface has the same P-gp inhibition effects. As we can see from Figure 6(B), at high concentration, both Bo-NP and Bo-loaded-NP can significantly increase the uptake of R-123. The Bo-NP better preserved the properties of Bo for it can also show a P-gp inhibition effect at middle and low concentrations. This better P-gp inhibition effect might be a possible reason for better cell uptake results of Bo-NP.

Oxidative stress has become the key harmful factor of brain ischemia/reperfusion (Fan et al., 2015). Ischemic brain injury leads to the overproduction of free radical (Ohsawa et al., 2007), which destroys cell membranes, DNA, and protein (Kumar et al., 2008) by inducing lipid peroxidation. The brain is particularly susceptible to free radical-mediated insults due to its high levels of unsaturated fatty acids and low levels of protective antioxidants (Wu et al., 2012; Yu et al., 2014). The SOD participates in the regulation of antioxidant defense by catalyzing superoxide anion disproportionation into H_2_O_2_ and O_2_ (Cherubini et al., 2005; Tao et al., 2013; Yun et al., 2013). MDA is an end product and a biomarker of lipid peroxidation (Xu et al., 2006; Liu et al., 2014). SOD activity and MDA content reflect the balance of oxidation and antioxidation in the brain. At 24 h after cerebral ischemia and reperfusion, MDA content was decreased and SOD activity was increased significantly by pretreatment with Bo-TSA-NP. Although the TSA-NP has certain effects on increasing the SOD content, it does not have effects on the MDA content.

TSA was proved with protective effects on CIRI by increasing SOD and decreasing MDA in serum after i.p. injection (Boban et al., 2009). In the current study, the protective effect of TSA on CIRI was significantly improved after IN delivery of Bo-TSA-NP. The brain delivery capacity of Bo modified NP enhanced the amount of TSA delivered into the brain. Thus, better improve the ability to scavenge oxygen free radicals and inhibit the degree of peroxidation of brain tissue in rats. The study done by Ye et al. (2021) showed the therapeutic effects of Bo modified TSA liposomes on cerebral ischemia reperfusion injury after intravenous injection. While in the current study, we explored the therapeutic effects of Bo modified TSA NPs on cerebral ischemia reperfusion injury after IN administration. Nasal administration is a noninvasive way of administration which may increase the patient compliances. We also compared the brain targeting performance of the NPs modified with Bo in different ways and optimized the formulation. It was found that Bo chemical modification was had a better brain targeting properties and its effects is probably based on the mechanism of inhibiting P-gp. In summary, the IN administrated Bo-TSA-NP which combined the brain targeting effects of Bo, IN delivered NPs can provide an ease and effective way for prevention of the CIRI.

## 5. Conclusions

In this study, we developed a novel Bo-TSA-NP for improving the brain delivery of TSA after IN delivery and enhancing the prevention effect of TSA on CIRI. This Bo-TSA-NP combined the advantages of the brain targeting property of Bo, the good drug loading effect of the NP with brain targeting property of nasal administration. The prepared Bo-NP demonstrated significantly improved uptake *in vitro* and protective effect of TSA on CIRI *in vivo*. This study provided a promising IN brain targeted delivery system for improve the CIRI prevention effects of TSA.
